# Investigating the impact of microbiome-changing interventions on food decision-making: MIFOOD study protocol

**DOI:** 10.1186/s40795-024-00971-6

**Published:** 2025-01-13

**Authors:** Meghedi Vartanian, Konrad Jakob Endres, Yee Teng Lee, Silke Friedrich, Marie-Theres Meemken, Imke Schamarek, Kerstin Rohde-Zimmermann, Robin Schürfeld, Lina Eisenberg, Anja Hilbert, Frauke Beyer, Michael Stumvoll, Julia Sacher, Arno Villringer, Julia F. Christensen, A. Veronica Witte

**Affiliations:** 1 Clinic for Cognitive Neurology, University of Leipzig Medical Center, Leipzig, Germany; 2 Department of Neurology, Max Planck Institute for Human Cognitive and Brain Sciences, Leipzig, Germany; 3 Department of Medicine III, Division of Endocrinology, Nephrology and Rheumatology, University of Leipzig, Leipzig, Germany; 4 Helmholtz Institute for Metabolic, Obesity and Vascular Research (HI-MAG), Helmholtz Center Munich at the University of Leipzig and the University Hospital Leipzig, Leipzig, Germany; 5 Integrated Research and Treatment Center AdiposityDiseases, Behavioral Medicine Research Unit, Department of Psychosomatic Medicine and Psychotherapy, University of Leipzig Medical Center, Leipzig, Germany; 6 Center for Mental Health, Helios Park Clinic, Leipzig, Germany; 7 Department of Cognitive Neuropsychology, Max Planck Institute for Empirical Aesthetics, Frankfurt/M, Germany

**Keywords:** Gut-Brain axis, Prebiotics, Neurocognitive behavioral intervention, Microbiome, MRI, Eating behavior, Obesity

## Abstract

**Background:**

Obesity is a multifactorial disease reaching pandemic proportions with increasing healthcare costs, advocating the development of better prevention and treatment strategies. Previous research indicates that the gut microbiome plays an important role in metabolic, hormonal, and neuronal cross-talk underlying eating behavior. We therefore aim to examine the effects of prebiotic and neurocognitive behavioral interventions on food decision-making and to assay the underlying mechanisms in a Randomized Controlled Trial (RCT).

**Method:**

This study uses a parallel arm RCT design with a 26-week intervention period. We plan to enroll 90 participants (male/diverse/female) living with overweight or obesity, defined as either a Waist-to-Hip Ratio (WHR) ≥ 0.9 (male)/0.85 (diverse, female) or a Body Mass Index (BMI) ≥ 25 kg/m^2^. Key inclusion criteria are 18–60 years of age and exclusion criteria are type 2 diabetes, psychiatric disease, and Magnetic Resonance Imaging (MRI) contraindications. The interventions comprise either a daily supplementary intake of 30 g soluble fiber (inulin), or weekly neurocognitive behavioral group sessions, compared to placebo (equicaloric maltodextrin). At baseline and follow-up, food decision-making is assessed utilizing task-based MRI. Secondary outcome measures include structural MRI, eating habits, lifestyle factors, personality traits, and mood. Further, we obtain fecal and blood samples to investigate gut microbiome composition and related metabolites.

**Discussion:**

This study relies on expanding research suggesting that dietary prebiotics could improve gut microbiome composition, leading to beneficial effects on gut-brain signaling and higher-order cognitive functions. In parallel, neurocognitive behavioral interventions have been proposed to improve unhealthy eating habits and metabolic status. However, causal evidence on how these “bottom-up” and “top-down” processes affect food decision-making and neuronal correlates in humans is still scarce. In addition, microbiome, and gut-brain-axis-related mediating mechanisms remain unclear. The present study proposes a comprehensive approach to assess the effects of these gut-brain-related processes influencing food decision-making in overweight and obesity.

**Trial registration:**

ClinicalTrials.gov NCT05353504. Retrospectively registered on 29 April 2022.

**Supplementary Information:**

The online version contains supplementary material available at 10.1186/s40795-024-00971-6.

## Introduction

The global obesity crisis, intensified by COVID-19, presents a major public health challenge, with far-reaching consequences for healthcare costs [1, 2]. In Germany, nearly a quarter of the population is living with obesity and more than half are overweight [3, 4]. Furthermore, studies have predicted an escalation in the prevalence of obesity in the ensuing decade if the current trend continues [5, 6]. This surge in obesity rates is related to environmental factors and dysregulated interactions between complex metabolic, hormonal, and neural mechanisms [7, 8]. The intricate, multifaceted nature of the disease, likely coupled with insufficient cross-disciplinary dialogue, impedes the efficacy of obesity treatments, resulting in an incomplete understanding of eating behavior [9].

Conservative treatments include behavioral and dietary weight loss counselling, which often do not induce significant weight loss in the long term [10]. Invasive treatments such as gastric bypass bariatric surgery demonstrate unparalleled efficacy and durability in weight loss, but only less than 50% of patients could maintain adequate surgery-induced weight loss on the long term [11–13]. In addition, obesity surgery sometimes goes along with unwanted effects such as nutritional deficiencies, fatigue, nausea, dry mouth, and constipation [14, 15]. Newly developed medication, e.g., Glucagon-Like Peptide-1 (GLP-1) agonists, offer clinically relevant weight loss and show high potential as treatment [15, 16], but are currently available for certain groups [10, 17, 18] with prolonged treatment periods, high costs [19] and weight regain after cessation [20]. A better understanding of underlying mechanisms of eating behavior could thus help to design novel preventive and complementary treatment options.

A positive energy balance due to increased caloric intake is likely one of the most important factors in accelerated weight gain [21]. Here, the role of the gut-brain axis in the regulation of appetite has attracted increasing research interest [22, 23]. The gut-brain axis serves as a bidirectional communication pathway between the gastrointestinal tract and the brain [22]. Through gut-brain signaling, various regulatory systems modify food decision-making and eating behavior [24, 25].

A so-called homeostatic system ensures the maintenance of energy balance and relies on physiological signals [26]. Here, the hypothalamus serves as a central hub integrating a multitude of peripheral signals from the gastrointestinal tract. These signals are then relayed through the brainstem, connecting the brain with the gut [27, 28]. Additionally, brainstem can further initiate eating-related motor actions such as chewing and swallowing [29]. In this intricate network, a pivotal region within the hypothalamus is the Arcuate nucleus (Arc). The Arc governs appetite through the interplay of appetite-stimulating and appetite-suppressing neuropeptides and hormones [30]. Interconnecting the Arc and the brainstem, the Paraventricular Nucleus (PVN) of the hypothalamus further orchestrates the regulation of energy balance during food intake to avoid overeating [31].

In the gastrointestinal tract, secreted hormones, peptides, and metabolites contribute to food intake regulation when reaching the brain via circulation or through vagus nerve stimulation. For instance, ghrelin, mainly secreted by the stomach, affects the hypothalamus, notably the Arc. It activates appetite-stimulating neurons while simultaneously suppressing appetite-suppressing neurons, thereby increasing appetite and inducing modifications in neural feeding circuits [32]. In contrast, Peptide YY (PYY), predominantly produced in the L-cells of the lower gastrointestinal tract, impacts neuronal activity in both the hypothalamus and brainstem. Specifically, PYY operates through Y2-receptors within the Arc, reducing food intake and promoting satiety by influencing the release of appetite-suppressing neuropeptides [33]. Similarly, GLP-1, derived from the pre-proglucagon gene expressed in pancreatic α-cells, intestinal L-cells, and specific neurons in the caudal brainstem and hypothalamus acts by delaying gastric emptying, reducing food intake, and influencing central feeding regulation [34]. Additionally, leptin and adiponectin, secreted by adipose tissue, inhibit appetite-stimulating neuropeptides and activate appetite-suppressing neuropeptides within Arc neurons, ultimately curbing food consumption [35–37]. In essence, this intricate interplay between hormonal and neural components, orchestrated mainly within the hypothalamus, serves as a central control hub for maintaining energy balance and regulating food intake.

Other aspects of eating behavior are often described as non-homeostatic, e.g., influenced by sensory cues, personal factors, and the rewarding aspects of food, shaping the perception of food pleasantness [38]. These signals are processed in regions including the Ventral Tegmental Area (VTA) and Nucleus Accumbens (NAc) [39], which comprise neuronal cell bodies or synapses building and secreting the neurotransmitter dopamine [40]. Repeated exposure to food cues heightens reward system activation [41]. This heightened activation is thought to lead to an amplified craving for highly enjoyable and pleasurable foods [42]. Dopamine- or other reward-related brain signals may also be involved in increasing the likelihood of recognizing and remembering the pleasantness or other features of food, which consequently might elevate consumption [43].

Meanwhile, eating high-calorie foods can reduce microbial diversity and richness in the gut [44–49] which could affect the different signals from the gastrointestinal tract by misregulating the release of peptides [50] or other metabolites and hormonal afferents. For instance, high-fat diets have been found to change fecal Short-Chain Fatty Acids (SCFAs) concentrations [51, 52] that may impact ghrelin-related signaling [50, 53] or lipid metabolism [54]. In addition, diets with added sugar reduced GLP-1 serum levels in a clinical trial by Jones et. al [55]. In turn, the fluctuations in the level of these hormones may further influence the activity of the brain’s reward system, especially the VTA and NAc [56]. This could impact non-homeostatic aspects of eating and improve the food reward experience [57–60].

Malfunctioning of the gut-brain axis signaling may thus affect homeostatic regulation of feeding and increase individual’s appetite or susceptibility to choose rewarding high-calorie foods. This contributes to additional weight gain in the long run and exacerbates the vicious cycle of overeating. Therefore, it can be hypothesized that targeting the gut-brain axis may offer novel intervention opportunities. On the one hand, a growing body of research shows how diet affects the gut microbiome composition [61], suggesting means to improve gut-brain signaling. Recently, in a proof-of-principle functional Magnetic Resonance Imaging (fMRI) study, we showed that a daily high-dosed prebiotic supplementary intake for 2 weeks compared to placebo reduced the brain response towards high-caloric food stimuli during food-decision making, potentially influencing dietary choices. This observed effect further relates to shifts in gut microbiota composition, notably the presence of bacteria capable of producing SCFAs [62]. However, the duration of the intervention period was relatively brief. In addition, evidence has centered on neurocognitive behavioral strategies that may improve unhealthy eating behaviors [63]. Nevertheless, there is a dearth of evidence on how these neurocognitive behavior strategies - often conceptualized as “top-down” processes - could also affect or be mediated through actions on the gut-brain axis and underlying neurobiological mechanisms. Therefore, this study seeks to investigate the impact of prebiotic and neurocognitive behavioral intervention on modifying eating behavior in obesity through the gut-brain axis in a medium-term (6-months intervention) RCT. We use high-resolution, non-invasive MRI techniques with accurate spatial and temporal insights into neural processes of food-related decision-making. We focus on the following main research questions in this trial:

1. Do prebiotic and/or neurocognitive behavioral intervention, in contrast to placebo condition, change food-related decision-making operationalized by task-based fMRI in people living with obesity?
2. Do prebiotic and/or neurocognitive behavioral intervention, in contrast to placebo, change food-related memory performance (encoding and retrieval), operationalized by task-based fMRI in people living with overweight and obesity?
3. Do prebiotic and/or neurocognitive behavioral intervention, in contrast to placebo change the gut microbiome (e.g., compositional changes and measures of diversity indices) and its related metabolites such as SCFAs?
4. Do prebiotic and/or neurocognitive behavioral intervention, in contrast to placebo, lead to modifications in brain structure, including white and grey matter volume, cortical thickness, and structural and functional connectivity?
5. Do the above-described intervention-induced changes in brain and cognitive markers relate to changes in markers of gut-brain signaling?
6. Do (a) metabolic markers, (b) high BMI, (c) lifestyle factors, (d) sex/gender, and (e) socioeconomic background/diversity predict alterations in both gut microbiome composition and brain structure and function?

## Methods

### Study design

**Fig. 1:**
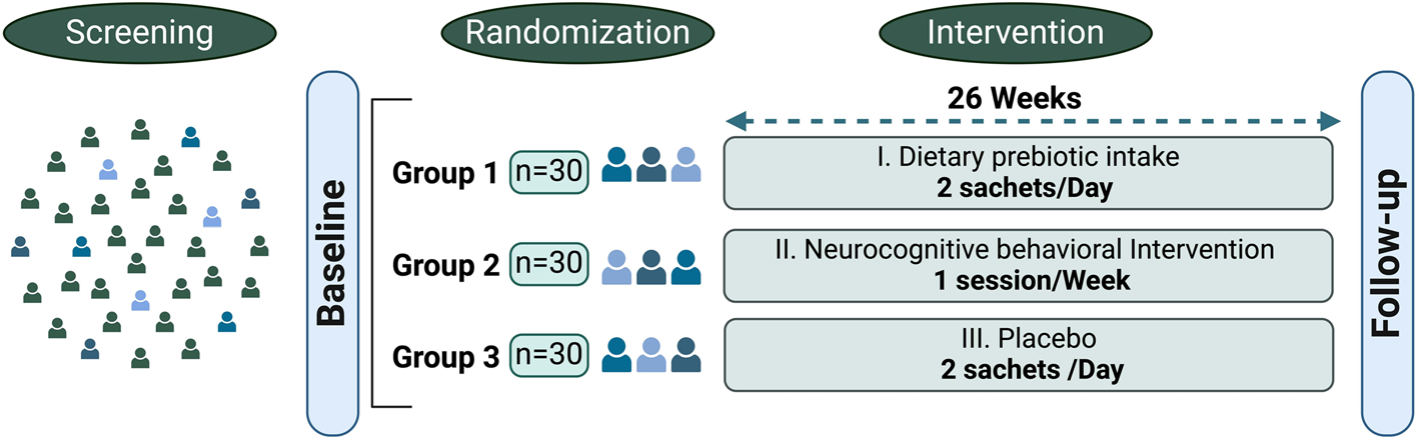
The study design. A total of 90 eligible participants will be allocated randomly to three groups after baseline measurement. Participants in the dietary prebiotic and placebo group receive 30 g inulin and equicaloric maltodextrin sachets, respectively, and are instructed to add these sachets to their regular diet twice a day. Participants in the neurocognitive behavioral intervention are instructed to attend weekly group-based sessions at the institute. All interventions span 26 weeks, with a follow-up measurement afterward. All baseline measures are repeated at follow-up assessments. All rights reserved ©BioRender

This study uses a 26-week, randomized controlled parallel design to examine the effects of prebiotic intake and neurocognitive behavioral intervention on food decision-making and the gut-brain axis. This is an ongoing clinical trial at the University of Leipzig in cooperation with the Max Planck Institute for Human Cognitive and Brain Sciences, Leipzig, Germany. It expands on a previous short-term study on prebiotics [62]. The study design is visualized in Fig. 1.

### Sample size estimation

**Fig. 2:**
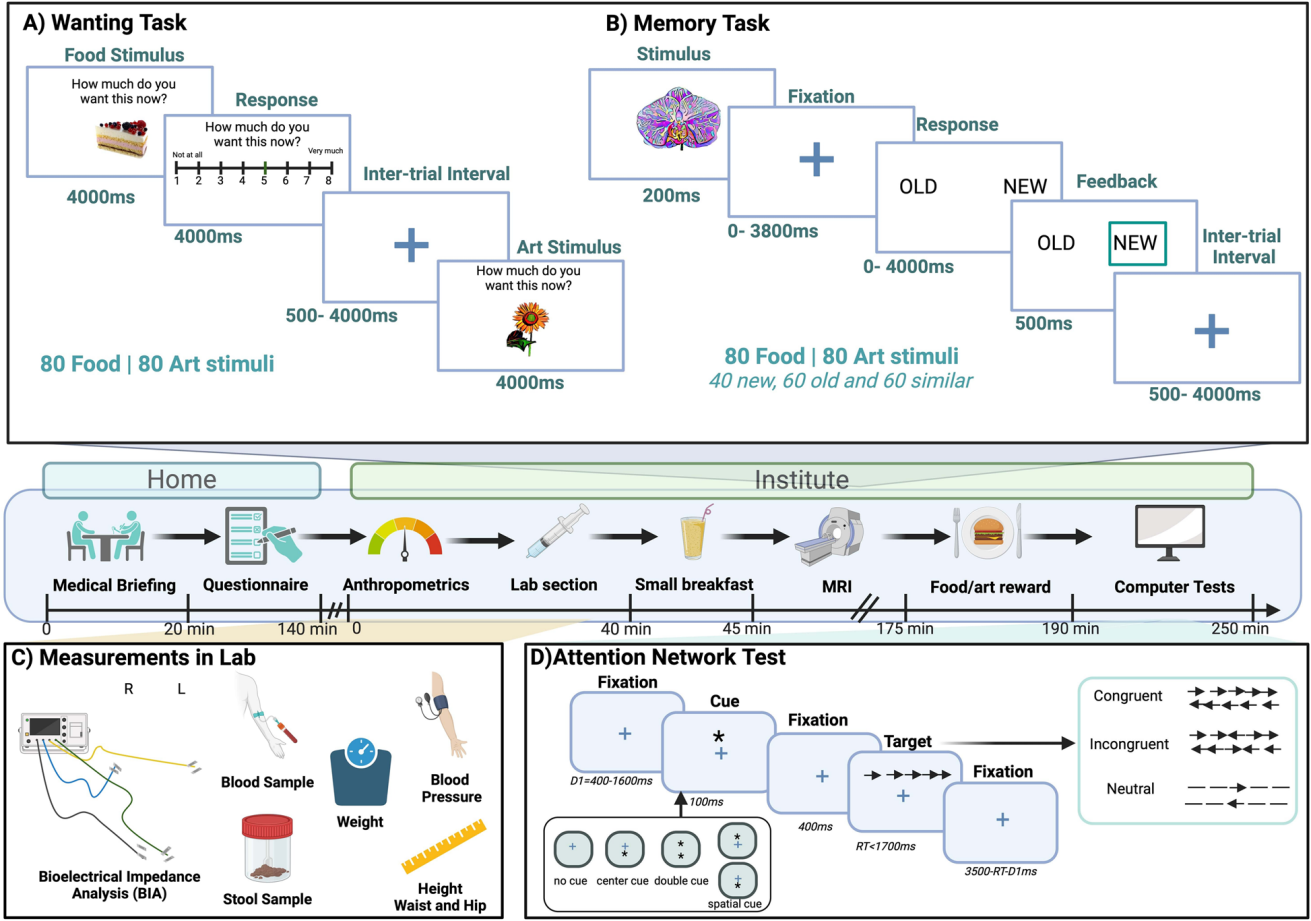
A summary of test-day procedure in baseline and follow-up. Upon arrival, feces samples are collected. Next, a blood sample is taken from participants in the fasting state to obtain various blood-based markers. Afterwards, blood pressure, anthropometrics, and body fat are measured. Subsequently, participants receive a small breakfast (10% of their daily energy need provided as a plant-based protein shake) and undergo MRI sequences including a food decision-making and a pattern separation memory task (for details, see sections A and B). The scan is paused after ca. 45 min to enable a second blood draw to monitor gastrointestinal hormone levels. When the scan is completed, participants are asked to consume their most wanted meal and receive a high-quality print-out of one of their top-rated art images and fill in another survey on-site. In the end, they complete a computer-based Attention Network Test (ANT) to measure executive function and alertness. The entire test day takes around 4–5 h. For each stage, we developed a Standard Operating Procedure (SOP) for reducing ambiguity and chances of human error. Participants are compensated with 12€ per hour for MRI and 10€ per hour for the remainder of the measurements. All rights reserved ©BioRender

To estimate effect sizes, we screened the literature and could not find directly comparable studies on prebiotic or neurocognitive behavioral intervention on food decision-making measured using task-based fMRI with a Likert scale. We inspected sample sizes in previous studies related to the topic. Two human studies reported changes in microbial composition due to a dietary change within 3–10 days in *n* = 11 and *n* = 22 participants, respectively [45, 64]. To inform a power analysis for a pairwise comparison between either prebiotic or neurocognitive behavioral intervention vs. placebo, we referred to previous results reported by Tiedemann et. al [65]. They examined the effects of intranasal application of insulin, and the comparison between insulin-resistance vs. non-resistance on reward rating response to food vs. non-food stimuli during task-based fMRI. Specifically, we chose the contrast reported in Fig. 2a (F [1, 46] = 5.49; *p* = 0.02, η2 = 0.12, *n* = 48, rmANOVA [65]), comparing to a small-to-moderate effect size of f = 0.37. This is also in the range or lower of the neural effects we reported in Medawar et al. after two weeks of prebiotics in a within-subject cross-over design [62]. According to outputs of the software G*Power [66] with a repeated measures ANOVA design to detect a significant difference of pre vs. post (2 measures) in the intervention compared to the placebo condition (2 groups). The calculation, with a power of 0.95, alpha of 5%, conservative zero correlation between measures, and no non-spheric correction, yielded a sample size of *n* = 50 for a pairwise intervention vs. placebo comparison. Including a second intervention group (*n* = 25) and estimating a 20% dropout rate, we aim to include in total of 90 participants.

### Participants

We aim to enroll 90 participants (m/f/d) living with obesity or overweight, defined as WHR ≥ 0.9 (m)/0.85 (f/d) or BMI ≥ 25 kg/m^2^ between the ages of 18 and 60 years. Exclusion criteria comprised the following: (a) Occurrence of psychiatric disease in the last 12 months (e.g., mood disorders, anxiety disorders, psychotic disorders, eating disorders, or substance abuse); (b) Any chronic inflammatory, malignant disease or untreated medical disorder reported in medical history; (c) Type 1 or 2 diabetes mellitus; (d) Previous bariatric/gastric surgery; (e) Non-correctable vision or hearing problems; (f) Contraindication to MRI; (g) Current pregnancy or breastfeeding (h) Medication that may affect appetite, weight and gut microbiota such as corticosteroids, oral contraceptives, antibiotics, and (i) Participating in weight loss programs or receiving non-invasive brain stimulation for the duration of the trial. Inability to follow the intervention instructions, defined by self-reported non-compliance in intake of > 50% of sachets or non-attention of 50% of the sessions, respectively, will also lead to exclusion. Also, volunteers who do not consent to get informed of incidental findings from MRI or blood measurements are ineligible to participate.

Eligibility is determined first by trained study staff during a telephone pre-screening and, secondly by in-house physicians during an in-person interview (MRI and medical briefing).

### Recruitment strategy

We started by recruiting participants from an internal database of individuals interested in participating in ongoing studies at the Max Planck Institute for Human Cognitive and Brain Sciences. In addition, we post study flyers on the recruitment webpage, and social media, or place them on openly accessible notice boards in the greater area of Leipzig, e.g., at supermarkets, pharmacies, etc. The study coordinator conducts phone pre-screening and provides study information to potential participants. Once they agree to participate, they receive the necessary written consent forms. The next step involves a screening examination to ensure that all inclusion criteria are met and none of the exclusion criteria apply. Upon successful recruitment, each participant is assigned a unique study ID.

### Blinding

During the initial screening, we inform participants that the study is investigating the impact of high-dose prebiotic (fiber) intake and neurocognitive behavioral intervention on eating behavior. Participants are randomized into three groups, blinded to differences in the dietary supplement groups. The study personnel responsible for the group randomization is not blinded and the sachets for the fiber and placebo groups are color-coded (Blue/Yellow) to avoid unblinding. Main investigators and participants are kept blinded to the allocation of study groups (sachets).

### Unblinding

We are permitted to reveal the participant’s intervention allocation (prebiotics or placebo) under certain conditions. These circumstances include situations where knowledge of the intervention allocation is essential for the treatment of a participant in a medical emergency, as well as those in which a participant may have experienced an unexpected serious adverse reaction. In this case, the data is regarded as unverified and will not be included in the analysis.

### Randomization

A weighted block randomization is used to balance the number of participants in the three groups that are run in parallel and to ensure the feasibility of the neurocognitive behavioral sessions. The random allocation sequence is generated using the randomizer website by group members not involved in data acquisition [67].

### Test-day procedure

We collect data both before and after the 26-weeks intervention. Prior to each test day, participants receive a feces sample kit via post and an email invitation to an online survey (questionnaires) to be completed at home. In addition, participants are required to fast for at least 12 h (overnight fast- water exception) and avoid any strenuous physical exercise. Figure 2 illustrates the summary of the test-day procedure.

### Feces and blood markers

Participants collect feces samples at home in a DNA/RNA shield fecal collection tube according to standardized instructions 48 h before test days. They are instructed to rate feces consistency using the Bristol stool scale [68] at home. The Bristol stool scale classifies feces into seven different types ranging from severe constipation (type 1) to severe diarrhea (type 7). The samples are frozen at − 80 °C. For the analysis of gut microbial composition, DNA extraction from the fecal samples will be performed, followed by Polymerase Chain Reaction (PCR) amplification of the hypervariable region V3-V4 of the 16 S rRNA gene. Then, amplicon-based Next-generation Sequencing (NGS) for 16 S rRNA gene and likely shotgun metagenomic sequencing will be utilized to determine the microbial profiles. We plan to send the samples to an external lab, e.g., Zytemo Research Inc. for analysis.

Blood samples are drawn after at least 12 h of a fasting period, and we ask participants to wear loose-fitting clothes and keep well-hydrated to facilitate the process. In addition, they are asked to avoid smoking, intensive physical activity, and stressful situations at least one hour before the start of the measurement. Furthermore, it is checked if they are a plasma/blood donor. The physician might advise an appropriate waiting period between blood donation and drawing based on individual health status.

The participants can sit or lie down for the blood draw according to their own preference. We then perform the blood draw and samples are kept at room temperature for a minimum of 25 min to allow clotting of serum samples before centrifugation at 3500U for 6 min and at 7 °C (thermo scientific Heraeus Labofuge 400 R centrifuge). After centrifugation necessary volumes for the analyses of pre-defined blood markers of both, serum and plasma are pipetted into separate 2 ml tubes, including up to 4 backups, to avoid freeze-thaw cycles and are stored at − 80 °C. The two whole blood samples and an RNA vacutainer are obtained in the EDTA K3E - Monovettes and the RNA vacutainer is directly frozen at − 80 °C for later use. For a second blood draw during the pause of the MRI acquisition, 2.5 ml blood serum is taken to check how the gastrointestinal hormones are fluctuating after the small breakfast intake (please see section “small breakfast”). Blood samples are used to measure the markers listed in Table 1. A total amount of < 45 ml of blood is taken during the test day. Where possible, leftovers of serum and full blood are stored as backup. We plan to send the blood samples to the Institute of Laboratory Medicine (ILM), Leipzig University, and external labs for less routine markers.

**Table 1:** Planned blood markers. For blood draw we use the following tubes: 3x S-Monovette serum gel CAT/9,0 ml (Sarstedt), 2x S-Monovette EDTA K3E/2,7 ml (Sarstedt), 1x S-Monovette fluoride EDTA FE/2,7 ml (Sarstedt), 1x Tempus Blood RNA tube (Thermo Fisher Scientific), 2x CAT serum sp clot activator Vacuette 2,5 ml (Greiner bio-one). Immediately after the blood collection, 25 µl of aprotinin protease inhibitor diluted 1:1000 in 0.9% NaCl is added to the test tube meant for the analyses of sensitive hormones such as PYY or Ghrelin for instance

Blood Markers	
**Glucose and Lipid Metabolism Markers**	- Glycated Hemoglobin [HbA1c] - Glucose - Insulin - Total Cholesterol - High-Density Lipoprotein [HDL] Cholesterol - Low-Density Lipoprotein [LDL] Cholesterol - Triglycerides - Leptin - Short-Chain Fatty Acids [SCFA]
**Inflammation Markers**	- Tumor Necrosis Factor Alpha [TNF-Alpha] - High-Sensitivity C-Reactive Protein [Hs-CRP] - Interleukin-6 [IL-6]
**Gastrointestinal Hormones**	- Ghrelin - Glucagon-Like Peptide-1 [GLP1] - Peptide YY [PYY]

### Blood pressure

We ask participants to sit in a relaxed and comfortable position for five minutes. The cuff is wrapped around the upper left arm, and it should be at the same height as the heart. For the measurement, we are using Omron M500 Intelli IT Upper Arm Blood Pressure Monitor with IntelliWrap Cuff (22–42 cm). We perform three repeated measurements, with a minimum of one-minute interval between measurements. The mean of all measures is taken forward for analysis.

### Anthropometrics and body fat

We measure BMI, WHR, and body fat percentage. Participants are asked to take off shoes and heavy clothing. The body weight and height are measured with precision, using a calibrated Seca robust 813 scale to the nearest decimal fraction for weight and a Seca 206 roll measuring tape for height. For the WHR, on top of the above-mentioned criteria, participants are asked not to hold their breath while measuring and the number on the tape measure right after exhale is recorded.

Body fat is assessed using the Bioelectrical Impedance Analysis (BIA) method with the BIACORPUS RX 4004 M device from Medical Healthcare GmbH, located in Karlsruhe, Germany. The measurements are processed using the Software Body Composition V9.xM- Version V9.0.21212–17 M – Professional.

BIA operates by passing a safe, low-level electrical current through the body using electrodes placed on the hands and feet. The inner electrodes are attached to the wrists and ankles at a distance of 3–5 cm from the outer electrode. As the current flows, it encounters impedance from different bodily tissues. Muscles and organs have low impedance because they are good conductors, whereas fat has a larger impedance since it contains less water. The components of body composition, such as Body Fat Mass (BFM) and Body Cell Mass (BCM), are then estimated by BIA devices using impedance measurements in conjunction with individual-specific data, such as height, weight, age, and gender.

### Small breakfast

Prior to the MRI scan, participants receive a protein shake (tasteless vegan protein with Oatly Haferdrink calcium vegan). This step is implemented to guarantee that participants do not feel satiated or hungry since the state of hunger can significantly impact brain activity during the fMRI scan. The amount of shake is based on individual basal metabolic rates calculated by the Harris-Benedict equation [69]. It takes into account the individual’s sex, weight, height, and age to estimate the needed calorie intake. For the preparation of the shake, a health certificate is acquired from the national health office in Leipzig, Germany.

### MRI data acquisition

Brain images are acquired using a 3 Tesla Siemens Magnetom Skyra MRI Scanner equipped with a 32-channel head coil. The following sequences are performed:

- Field maps and ap/pa are acquired to be used for correcting scanner inhomogeneity in the preprocessing pipeline.
- T1-weighted MPRAGE sequence that provides detailed structural information about the tissues and anatomical structures using the ADNI protocol with the following parameters: TR = 2300 ms; TE = 2.98 ms; flip angle = 9°; FoV read: 256 mm; voxel size: (1.0 mm) ³; 176 slices.
- Diffusion-weighted Tensor Imaging (DTI) sequence that is utilized primarily to investigate the microstructural features of tissues, especially the brain’s white matter structures. DTI is particularly effective for evaluating the integrity and direction of nerve fibers in the brain as it is sensitive to the movement of water molecules inside tissues with the following parameters (TR 6000 ms; TE 80 ms; TI 2500 ms; flip angle 90°; FoV read 220 mm; voxel size: (1.7 mm)³; 88 slices; max. b = 1000 s/mm² in 60 diffusion directions (+ 7 b0-images); partial Fourier = 7/8; GRAPPA-factor = 2; interpolation = OFF. Ap/pa-encoded b0-images are acquired for distortion correction.
- Task-based fMRI is acquired using T2*-weighted images used to investigate how the brain responds to specific cognitive tasks or stimuli with the following parameters EPI BOLD: repetition time TR = 2000 ms, TE = 23.6 ms, flip angle = 80 °C, FoV read = 204 mm; voxel size 2 × 2 × 2 mm^3^; 60 slices; slice thickness 2.00 mm, gap 0.26 mm; orientation T > C −15°; multi-band = 3, interleaved, 950 measurements.
- Fluid-attenuated Inversion Recovery (FLAIR) sequence is used to enhance the visibility of pathological tissues in the brain with the following parameters (TR 10000 ms; TE 90 ms; TI 2500 ms; flip angle 180°; slice thickness 4.00 mm; FoV read 220 mm).

The paradigm lasts approximately 100 min including a break in between. Finally, participants fill in a feedback form on their experience in the scanner.

### fMRI task

The imaging procedure comprises two fMRI tasks, each of which has a duration of around 30 min (Fig. 2, A-B).

A) Food wanting: We use an event-related design with standardized fMRI task of food [70] and art stimuli [71]. Each run contains 160 stimuli (80 food and 80 art). Food stimuli include sweet, savory, processed, whole foods and beverages. Art stimuli include three groups of animals, objects, and plants each in four different art styles (“Azulejos,” “Klimt,” “Munch,” and “Pointillism” or “Dalí,” “Hundertwasser,” “Picasso,” and “Popart”). Each image is presented only once and for baseline and follow-up measurements, different stimuli sets are being used. The duration of each trial is 4000 ms followed by a 4000 ms pause for wanting rating of the stimulus with 500–4000 ms intervals between trials. Briefly, participants are asked during scanning to rate their desire (on an 8-point Likert scale) to eat the presented food or to receive the art item as a print following the MRI session. The order of stimuli and direction of scale (forward or reverse) are randomized for participants. Participant’s subjective hunger ratings are recorded at the beginning and end of the task. The initial position of the rating cursor is randomized to avoid motor artifacts related to specific rating.
B) Food memory: In this fMRI task, there are 160 stimuli (80 food and 80 art) of which there are 40 new, 60 old, and 60 similar. The old stimuli were previously presented in the wanting task. The new stimuli are presented for the first time in the food memory task. The similar stimuli closely resemble those shown before (art stimuli have a different style but the same image, while food stimuli are new but match in content). The duration of each trial is 500 ms followed by a 4000 ms fixation cross. Next, participants are asked if the stimulus shown is “new” or “old” for 0–4000 ms. The color of the selected answer is changed as feedback with 500–4000 ms intervals between trials.

### Questionnaires

There are two sets of questionnaires in the Lime Survey. A psychologist checks the institute set (with clinical questionnaires) immediately after completion, to facilitate necessary action in case of clinical emergency (e.g., suicidal thoughts). The list of questionnaires for the “institute” and “home” sets is stated in Table 2.

**Table 2:** List of questionnaires used in the study. Questionnaires are categorized based on whether they are filled out at the institute or at home. The “institute set” lasts around 15 min, and the “home set” lasts around 2 h. for the questionnaires a license is issued from Hogrefe Verlag GmbH & Co. KG. All data transfers are encrypted in Lime Survey, and no personal data is transferred to third parties

Sets	Questionnaires
Home	- General Questionnaire [62] - State of Health (*SF36*) [72] - State Trait Anxiety Depression Inventory (*STADI S* and *STADI T*) [73] - The revised NEO Personality Inventory (*r-NEO*) [74] - Three Factor Eating Questionnaire (*TFEQ*) [75] - Food Craving Questionnaire- trait (*FCQ-tr*) [76] - Power of Food Scale (*PFS*) [77] - Food Frequency Questionnaire (*FFQ*) [78] - Yale Food Addiction Scale (*YFAS*) [79], - Sleep Questionnaire (*SF/BR*- last two weeks) [80] - The International Physical Activity Questionnaires (*IPAQ*) [81] - The Gastrointestinal Quality of Life Index (*GIQLI*) [82] - The Perceived Stress Scale (*PSS*) [83] - Positive and Negative Affect Schedule (*PANAS*) [84] - Behavioral Inhibition System (*BIS*) - Alcohol Use Disorders Identification Test (*AUDIT*) [85]
Institute	- Sleep Questionnaire (*SF/AR*- last night) [80] - Beck Depression Inventory (*BDI*) [86] - General Depression Scale (*GDS*) [87] - Eating Disorder Examination Questionnaire (*EDE-Q*) [88] - Profile of Mood States (*POMS*) [89]

### Attentional network test

The ANT is designed to evaluate three distinct attentional networks: alerting, orienting, and executive control. During the task, participants are typically presented with a central target stimulus flanked by distractors. They are instructed to respond based on specific rules or conditions, such as indicating the direction of an arrow while ignoring distractors. The reaction time and accuracy of the participant’s responses are measured to provide insights into their attentional capabilities and efficiency [90]. The task contains three experimental blocks, each consisting of 96 trials, and lasts around 20–30 min (for details, see Fig. 2, D).

## Interventions

### Dietary prebiotic intervention

Participants receive dietary prebiotic supplements for a duration of 26 weeks (Orafti Synergy1 contains approximately 92 ± 2 g of inulin and 8 ± 2 g of glucose, fructose, and sucrose per 100 g). The supplements are self-administered as a powder in 2 sachets (2 × 15 g) per day in addition to the participant’s diets. Participants are recommended to start with only one sachet per day in the first week of the intervention to reduce the risk of gastrointestinal complaints and adverse reactions.

### Neurocognitive behavioral intervention

**Fig. 3:**
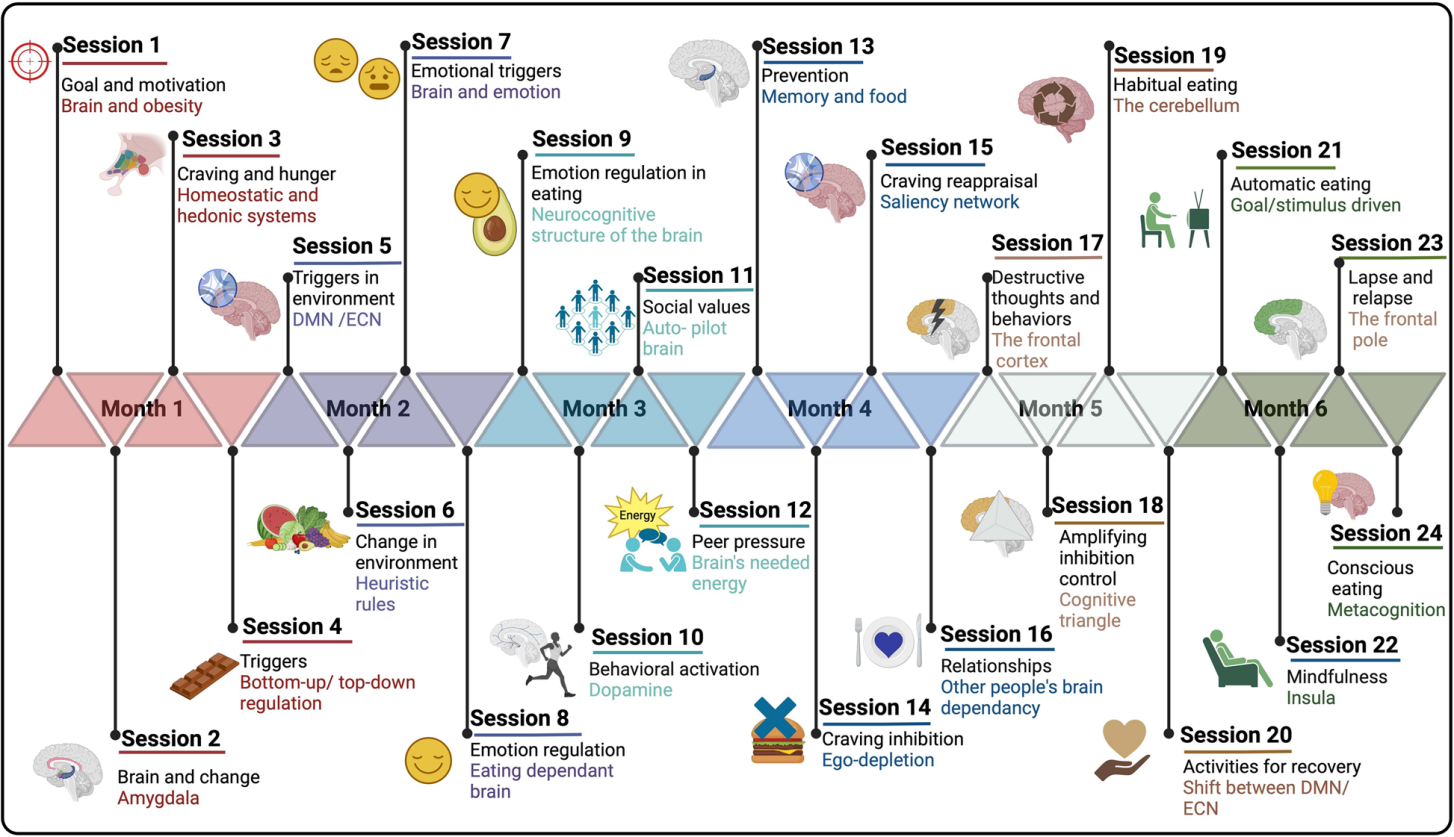
An overview of the content structure for the neurocognitive behavioral intervention sessions. Each session adheres to a consistent format, while its content varies, focusing on dimensions that influence (over)eating. The session begins with an educational segment, where participants gain insight into the intricate connection between their eating behaviors and brain function. Subsequently, real-life examples are discussed, encouraging participants to share their own experiences, facilitated by the mediator. Next, cognitive, and behavioral strategies are introduced, enabling participants to implement these approaches in their daily lives and tailor them to their specific needs. Each participant is provided with worksheets to practice the session’s content throughout the week and document the challenges they encounter. All rights reserved ©BioRender

The neurocognitive behavioral intervention is inspired by the book “Brain Obesity: Practical Neuroscience-Informed Methods to Keep Your Body Fit and Healthy” [91]. It encompasses weekly sessions, each with a duration of approximately one hour, extending over a six-month period. These sessions are conducted in a group setting and facilitated by a trained mediator. The module presented in the book follows a comprehensive model considering the various dimensions of overeating. These sessions are held in a group of usually 7–9 participants in a hybrid format (see Fig. 3 for more detail).

### Placebo

Participants receive placebo supplements for 26 weeks, in an equicaloric amount to the prebiotic inulin (Maltodextrin DE 19, a spray-dried starch saccharification product resulting from maize starch hydrolysis, contains 95 g of carbohydrates, including 8 g of sugars). The process of taking the placebo supplements is identical to the prebiotic arm.

### Analysis plan

A comprehensive report, encompassing in-depth hypotheses for each primary research question, the corresponding analysis strategy, and the associated statistical codes, will be accessible through OSF.io as a sub-project stemming from the main “MIFOOD” project [92].

## Discussion

In this RCT, we aim to test whether a prebiotic and/or neurocognitive behavioral intervention, compared to placebo, will improve food decision-making, measured using task-based fMRI, in adults living with overweight and obesity. In addition, we aim to shed light on intervention-induced changes in gut-brain communication pathways that could underly or accompany these effects.

To this end, participants undergo task-based and structural high-resolution MRI, donate feces and blood in (semi-) fasted state, and respond to additional tasks and questionnaires of a diverse array of biomarkers from the microbiome, blood, and psychological domains. The overall goal is to create a comprehensive and resourceful longitudinal database of the brain and gut response to microbiome-changing interventions in individuals at risk of or with obesity. This will promote evidence-based prevention and treatment strategies and broaden our understanding of therapeutic improvements.

Research targeting the impact of a healthy diet on the gut microbiome, particularly influenced by fiber from fruits, vegetables, and other plant-based foods is growing [93, 94]. Studies have shown that intake of inulin, a source rich in prebiotic soluble fiber found for example in wheat or chicory, increases the abundance of Actinobacteria and Bifidobacteria, which are producers of SCFAs [95]. SCFAs such as acetate, propionate, and butyrate are the main byproducts of fiber fermentation which may cause a wide range of physiological consequences from ecological to direct metabolic and immunological effects on the host [96].

Indeed, it has been suggested that SCFAs are also involved in gut-brain axis crosstalk. SCFAs may also exert an impact on the mucosal immune system and modulate the homeostatic pathway [97, 98], which later influences information processing in the Central Nervous System (CNS) [99]. In one animal study it was found that particularly butyrate, reduced appetite and hypothalamic neuronal signaling, which further reduced fat mass gain and prevented obesity [100]. Besides, a randomized crossover study showed that an increased level of propionate was associated with decreased Blood Oxygenation Level Dependent (BOLD) signal in the caudate and NAc among 20 healthy men during high-energy food picture evaluation, which signified the role of propionate in modulating eating behavior via striatal pathways [101]. In a recent study of our group, participants who received prebiotics compared to maltodextrin had higher levels of SCFA-producing Bifidobacteriaceae and showed less activation in the right orbitofrontal cortex and ventral tegmental region of the brain in response to high-caloric desired food stimuli [62]. In other words, specific nutrients and synergistic nutrient patterns may impact “bottom-up” signaling and thereby potentially modify brain function and eating behavior.

In a year-long study of two individuals, daily measurements revealed that common human actions swiftly altered stable microbial communities. For instance, changes in dietary fiber intake correlated with shifts in approximately 15% of gut microbiota members the following day [102]. However, if the consumption of the above-mentioned food sources is interrupted, the reported microbial changes disintegrate during a 21 to 28 days wash-out [103, 104]. A short time frame thus appears insufficient to accurately assess a sustained maintenance of dietary change which is necessary in eating habits. Therefore, we propose conducting further investigations to enhance our comprehension of the physiochemical characteristics of dietary fiber and its connection to eating behavior in an extended duration of 26 weeks as the availability of high-quality human experimental studies remains limited.

In parallel, Cognitive Behavioral Therapy (CBT) has evolved through different theoretical approaches to the effectivity of cognitive processes on behavior change [105], eventually leading to the novel concept of neurocognitive behavior interventions as presented in the current study. The initial CBT waves were mainly focused on classical learning theory which explored how foods could trigger psychological and physiological reactions, priming the body for consumption [106]. Subsequently, operant learning theory was embraced, which described how individuals learn from the outcomes of their food-related decisions by seeking rewards and avoiding punishments [107]. The approach later evolved to integrate coping and social learning theory, where food intake is modified in response to a variety of parameters, such as eating partners, gender, body weight of the partner, and personal characteristics [108]. Finally, self-control strategies, such as self-monitoring were integrated which demonstrates a consistent association with successful weight management [109]. In sum, the main focus of the theories and strategies in the initial waves has been educating individuals and modifying environmental cues to induce alteration in dietary and physical activity [110].

During intermediate waves, beyond environmental changes, cognitive training, primarily rooted in attribution and cognitive theories, centered on the intricate aspects of how an individual engages with their internal experiences of an event, rather than the event itself [111]. For instance, considering the negative consequences of eating unhealthy food can reduce the reported craving [112], or highlighting a food’s health benefits can help individuals make healthier decisions [113, 114]. These strategies were well integrated into behavioral strategies, forming the foundation of CBT, which has had a significant influence on eating behaviors to date [115].

Nevertheless, the subsequent waves of CBT appeared in an attempt to increase the effectiveness of the first and second waves by emphasizing the influence of these attributions on emotions, expectations, and future actions [116]. In this context, cognitive reappraisal of unpleasant emotions could help improve food choices, particularly favoring nutritious options [117, 118]. In addition, emotions tied to eating can be regulated through mindful eating when attention is redirected from strict dietary guidelines to experiences in the present, enabling people to make conscious food choices [119].

Leveraging the insights we have gained from cooperative dynamics in CBT waves, the neurocognitive behavioral intervention now orchestrates a multi-dimensional, integrated approach along with enhancing individual’s eating-related brain literacy with the objective of fostering the adoption of a health-conscious lifestyle – “top-down“ [120, 121]. Moreover, multidisciplinary studies suggest that cognitive therapies may well inform gut-brain signaling through multiple routes including altered Hypothalamic Pituitary Adrenal (HPA) axis, food intake, gut motility, and stress response. All these routes might also affect microbiota and SCFA signaling and subsequent brain signaling [122, 123]. The current study will thus help to understand the effects of the intervention on neural correlates of food decision making, in addition to possible effects on the gut-brain axis and related functional implications.

As for the placebo intervention, maltodextrin is considered a safe substance that resembles inulin in taste and appearance [124] without the active components present in the prebiotic intervention [125]. In addition, it acts as an active control group for neurocognitive behavioral intervention, which is preferable to an inactive waiting list group [126] or additional group-based placebo [127, 128] as the blinding is maintained by this procedure for the placebo group. The other downside of an additional group-based placebo is psychological treatment rationale, as it is essential that the content naturally correlates with eating behavior to keep the participant’s adherence. Concurrently, it is crucial to ensure that the thematic content does not overlap with the module addressed within the intervention group. Moreover, the thematic material must be firmly based on empirical evidence; otherwise, ethical limits may apply to the permissibility of the content.

Considering common limitations in dietary- and behavioral intervention RCTs, we consider that participant’s compliance with the intervention or follow-up assessments can affect the quality and reliability of the data. High dropout rates might be probable in the course of six months and can introduce bias and impact the statistical power of the study. To enhance adherence, we gradually increased the dosage for the sachets group, trying to minimize gastrointestinal complaints while still reaching the 30 g target. We ensured ongoing support by keeping regular contact with participants via phone and email and gave them a calendar to monitor their daily intake. In the neurocognitive behavioral group, we placed a strong emphasis on scheduling flexibility, offering hybrid mode to accommodate diverse needs effectively.

In sum, with this multidisciplinary study approach, we would like to highlight the multifaceted nature of eating behavior and challenge the oversimplified conception that an individual’s motivation or compliance solely upstands food intake. Instead, we underscore the significant contributions of biological mechanisms, such as changes in gut hormone secretion, metabolic changes, and the brain’s networks involved in appetite and craving. Understanding the convoluted nature of these processes and their response due to novel interventions will eventually help to develop tailored, comprehensive long-term weight management strategies.

## Supplementary Information

Supplementary Material 1.

Supplementary Material 2.

## Data Availability

No datasets were generated or analysed during the current study.

## Abbreviations

ANT: Attention Network Test
ARC: Arcuate Nucleus
AUDIT: Alcohol Use Disorders Identification Test
BCM: Body Cell Mass
BDI: Beck Depression Inventory
BFM: Body Fat Mass
BIA: Bioelectrical Impedance Analysis
BIS: Behavioral Inhibition System
BMI: Body Mass Index
BOLD: Blood Oxygenation Level Dependent
CBT: Cognitive Behavior Therapy
CNS: Central Nervous System
DTI: Diffusion-weighted Tensor Imaging
EDE-Q: Eating Disorder Examination Questionnaire
FCQ-tr: Food Craving Questionnaire- trait
FFQ: Food Frequency Questionnaire
FLAIR: Fluid-attenuated Inversion Recovery
fMRI: functional Magnetic Resonance Imaging
GDS: General Depression Scale
GIQLI: Gastrointestinal Quality of Life Index
GLP-1: Glucagon-Like Peptide-1
HbA1c: Glycated Hemoglobin
HDL: High-Density Lipoprotein
HPA: Hypothalamic Pituitary Adrenal
Hs-CRP: High-Sensitivity C-Reactive Protein
IL-6: Interleukin-6
ILM: Institute of Laboratory Medicine
IPAQ: International Physical Activity Questionnaires
LDL: Low-Density Lipoprotein
MRI: Magnetic Resonance Imaging
NAc: Nucleus Accumbens
NGS: Next Generation Sequencing
PANAS: Positive and Negative Affect Schedule
PCR: Polymerase Chain Reaction
PFS: Power of Food Scale
POMS: Profile of Mood States
PSS: Perceived Stress Scale
PVN: Paraventricular Nucleus
PYY: Peptide YY
RCT: Randomized Control Trial
r-NEO: revised- Neo Personality Inventory
SCFA: Short-Chain Fatty Acids
SF/AR-BR: Sleep Questionnaire- "Schlaffragebogen" in German
SF36: Short Form - State of Health
SOP: Standard Operating Procedure
STADI S: State Anxiety Depression Inventory
STADI T: Trait Anxiety Depression Inventory
TFEQ: Three Factor Eating Questionnaire
TNF-Alpha: Tumor Necrosis Factor Alpha
VTA: Ventral Tegmental Area
WHR: Waist to Hip Ratio
YFAS: Yale Food Addiction Scale
