# Supplementary material for: Investigating the impact of microbiome-changing interventions on food decision-making: MIFOOD study protocol
Source: BMC Nutr. 2025 Jan 13;11:8. doi: 10.1186/s40795-024-00971-6 (PMC11727427; doi:10.1186/s40795-024-00971-6)
Supplement: Supplementary file 2 — Supplementary Material 2. [file 40795_2024_971_MOESM2_ESM.pdf]

General Questionnaire- MIFOOD

1. Which gender do you identify yourself with?
    - o Male
    - o Female
    - o Diverse

2. Please enter your date of birth.

DD.MM.YYYY [ ]

3. What is your **main job**?
    - o Full time employed
    - o Part-time employed
    - o Occasionally or irregularly employed
    - o Exclusively in retirement/pension
    - o Studying
    - o Unemployed

4. What is your average household income?
    - o Less than €500
    - o 500 - 1000 €
    - o 1000 - 2000 €
    - o 2000 - 3000 €
    - o 3000 - 4000 €
    - o 4000 - 5000 €
    - o More than €5000

5. How do you rate your health in general?
    - o Very good
    - o Good
    - o Mediocre
    - o Bad
    - o Very bad

6. Are you left-handed or right-handed?
    - o Left-handed
    - o Right-handed
    - o Both

7. Do you smoke? If yes, in what quantities?
    - o No
    - o Yes

Please enter your comment here:

8. Do you regularly consume alcohol? If yes, in what quantities?

- o No
- o Yes

Please enter your comment here:

9. Have you used cannabis, amphetamines, cocaine, ecstasy, barbiturates, tranquilizers, opiates, psychedelics or similar drugs within the last month? If yes, which?
   - No
   - Yes

Please enter your comment here:

10. Are you pregnant, breastfeeding or planning to become pregnant in the next 5 months?
    - No
    - Yes

Please enter your comment here:

11. Would you describe yourself as a competitive athlete? (If yes, to what extent? How often and for how long do you do sport? What sport?)
    - No
    - Yes

Please enter your comment here:

12. Please enter your body weight (in kg)

13. Please enter your height (in cm)

14. Do you drink more than 6 cups of coffee a day?
    - No
    - Yes

15. Are you taking the pill to prevent pregnancy?
    - No
    - Yes

Please enter your comment here:
